# *In Vivo* Effects of Antiviral Protein Kinase C Modulators on Zebrafish Development and Survival

**DOI:** 10.5402/2011/248280

**Published:** 2011-12-20

**Authors:** Richard V. Davis, Lisa N. McKernan, Jennifer Rhodes, Joseph Kulkosky

**Affiliations:** ^1^Department of Biology, Chestnut Hill College, Philadelphia, PA 19118, USA; ^2^Fox Chase Cancer Center, Philadelphia, PA 19111, USA

## Abstract

Clinical interventions using protein kinase C (PKC) modulators have been proposed for eradication of HIV-1-infected cellular reservoirs which persist in patients despite prolonged antiretroviral therapy. The effects of some of these agents have not been assessed in a developing vertebrate model. This study examines the developmental and toxicological effects of these compounds on zebrafish embryos and larvae. Treatment of zebrafish through the first week of development with various PKC pathway modulators did not elicit gross physical defects or elevated incidences of death at lower doses. Higher concentrations resulted in rapid death for both later-stage embryos and larvae. Each compound had a threshold dose for lethality. The defined nonlethal doses may be useful toward assessing the effects of modulating PKC activity on zebrafish development. They may further provide some guidance for the potential dosing of PKC modulators in clinical trials toward the goal of HIV-1 reservoir eradication.

## 1. Introduction

There has been considerable interest in the possibility that the eradication of persistent viral reservoirs in HIV-1-infected patients could be achieved through specific upregulation of viral expression from quiescently infected reservoir cells [1–6]. These silent viral reservoirs, largely comprised of HIV-1-infected, resting CD4+ T cells, are long-lived despite continuous and lengthy administration of HAART or antiretroviral therapy [3, 4, 7]. Eradication of these persistent reservoirs may be possible if a sufficient level of viral expression could be induced from the latent proviruses in order to trigger immune clearance or apoptosis of infected reservoir cells [3–6]. A number of diverse agents upregulate viral transcription from latent HIV-1 proviral templates *in vitro* and *in vivo*. These compounds include small chemical cellular activators such as prostratin, a member within the phorbol ester family, the macrolide lactone bryostatin-1, chromatin-remodeling agents, and select cytokines (primarily interleukins and interferons) [1, 5, 8–12].

Upregulation of latent HIV-1 expression via the phorbol ester family of compounds or bryostatin-1 occurs as a consequence of activating or modulating the protein kinase C pathway [2, 8, 9, 11, 12]. The ability of such compounds to upregulate HIV-1 transcription has been well documented in several cellular systems including the latently infected U1 or ACH 2 cell-lines, primary T lymphocytes from HAART patients as well as the scid-hu mouse model for HIV-1 infection [2, 8]. The concentration ranges of the PKC activating agents investigated in this study effectively upregulate latent HIV-1 expression in primary cells of infected patients [2]. However, developmental and toxicological effects associated with administration of these agents in an intact whole animal model have not been thoroughly evaluated.

 One purpose of this study was to rapidly assess the gross effects of PKC activation or modulation on zebrafish embryos and larvae, particularly morbidity or lethality, as major indicators of whether nontumor promoting phorbols, including prostratin, or the lactone bryostatin-1, could be regarded as serious candidates for use in humans at reasonable and effective doses. The tumor-promoting property of some phorbol esters essentially excludes these agents from consideration for clinical administration. The observation that zebrafish are able to uptake PKC activating agents from the media also provides impetus to observe the effects of modulating protein kinase C pathway activity on zebrafish tissue formation and function [13].

Modulation of the PKC signal cascade can have widely diverse effects upon cells and whole tissue systems. Notable effects, apart from the potential of HIV-1 reservoir eradication in humans, include the apparent extension of memory which may bear relevance toward the treatment of Alzheimer's disease as well as the amelioration of certain malignancies [14–16].

## 2. Materials and Methods

### 2.1. Zebrafish Strains

Wild-type AB strain and Fli-1 transgenic strain zebrafish were maintained in separate tank systems for embryo and larvae harvesting. Fli-1 transgenic zebrafish bear an enhanced green fluorescent (eGFP) open reading frame linked to the Fli-1 promoter which drives zebrafish expression of the eGFP transgene in blood vessels.

### 2.2. Embryo Treatment

Embryos a few hours after fertilization were distributed into 60 × 15 mm sterile culture dishes containing equal volumes of embryo media comprised 5 mM NaCl, 0.17 mM KCl, 0.33 mM CaCl_2_·2H_2_O, and 0.33 mM MgSO_4_·7H_2_O. Typically, each dish contained 40–50 embryos per dish then treated once daily by replenishing the media with the concentrations of agents as stated for three consecutive days or as stated otherwise. Samples included a negative vehicle control, 1% dimethyl sulfoxide (DMSO) and varying concentrations of prostratin or a phorbol-12 myristate-13 acetate (PMA) positive control at 10 uM, each suspended in DMSO. The treated embryos were then observed for developmental abnormalities or mortality using fluorescent or light microscopy. Specimens were observed and photographed under blue or visible light at times as indicated. Fluorescent images under blue light were captured using an Olympus CK40 fluorescent microscope equipped with a digital camera. Visible light photos were obtained using a Fujifilm Finepix A610 digital camera with the lens placed onto one eyepiece of dissecting microscope bearing an illuminated specimen base.

### 2.3. Larvae Treatment

Hatched larvae were evenly distributed into 60 × 15 mm sterile culture dishes at a minimum of 20 larvae/dish containing embryo media. The larvae were treated once with the concentrations of agents as indicated and harvested 60–75 minutes after treatment then stored at –70 degrees C for subsequent PAGE/Western analysis. Typically, fourteen larvae from each treated population were harvested by transfer into individual 1.5 mL microfuge tubes on ice using 1 mL plastic transfer pipets. The remaining specimens remained in the media and were photographed either immediately or 24 hours after treatment using 0.4% tricaine as an anesthetic for surviving specimens.

### 2.4. PAGE and Western Blot Analysis

Zebrafish specimens harvested and frozen at −70°C were thawed and suspended in 50 uL of Laemmli protein loading buffer (Biorad, Hercules, CA, USA). Samples were homogenized by hand using a plastic mortar for microcentrifuge tubes. Equivalent quantities of protein were loaded to each lane, quantified by using the Lowry Protein Assay (Biorad, Hercules, CA, USA). Prior to gel loading, the samples were heated to 90°C for 5 minutes and centrifuged at 12,000 ×g for 3 minutes. The supernatants were removed for SDS-PAGE. Samples were electrophoresed on 4%–15% gradient gels (Biorad, Hercules, CA, USA) and proteins transferred to PVDF membranes using Trans-Blot Semi Dry Transfer Cell (Biorad, Hercules, CA, USA) at 15 V for 1-2 hours. Protein blots were incubated in the presence of anti-GFP antisera (Bio-Rad, Hercules, CA, USA) or anti-MAPK-8 zebrafish-specific antisera obtained from Anaspec, Freemont, CA, USA. PVDF membranes were developed using the WesternBreeze Chromogenic Western Blot Immunodetection Kit as per instructions using anti-rabbit IgG conjugated with alkaline phosphatase. Images of blots were captured by scanning with an HP Officejet PRO combination printer/scanner.

## 3. Results

### 3.1. PKC Modulators Do Not Affect Early-Stage Zebrafish Embryo Development

**Figure 1:**
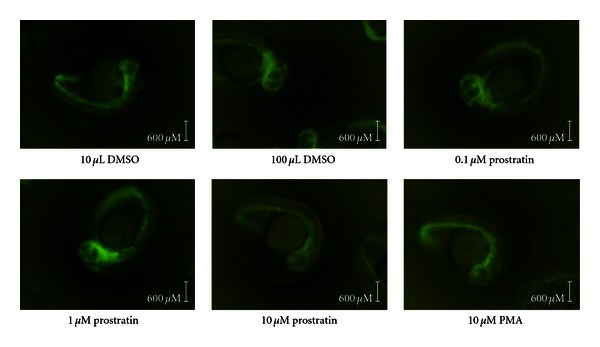
Prostratin and PMA do not alter embryonic development of zebrafish embryos. Day-1 postfertilized embryos were treated with concentrations of agents as indicated. Fluorescent images of the treated embryos were captured 24 hours later on Day-2 postfertilization.

As shown in Figure 1, no abnormalities or increased mortality were observed one day after treatment of day 1 postfertilization embryos with varying concentrations of prostratin and 10 uM PMA compared to embryos treated with an equivalent concentration of the control vehicle, DMSO. The development of the embryonic vascular systems appeared similar in all treated samples as assessed by eGFP fluorescence of vascular endothelial cells which are visibly highlighted via expression of the Fli-1 promoter/eGFP transgene. The eGFP fluorescence notably highlights the vascular endothelium in the anterior head portion and extending down the developing vertebral column of the embryos in these images.

**Figure 2:**
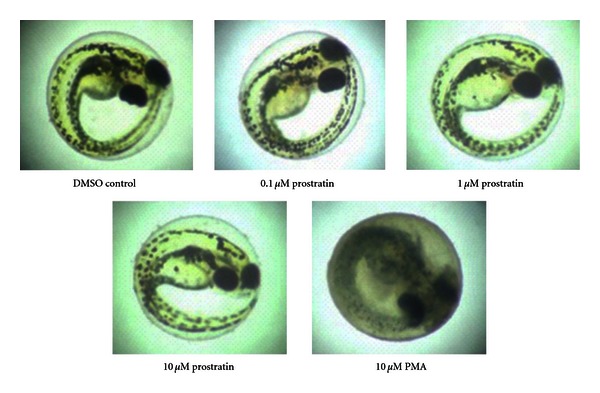
Similar concentrations of prostratin and PMA have differential lethal effects on embryos. Embryos were treated daily for 3 days with the agents at concentrations as indicated and photographed 12 hours later.

 As shown in Figure 2, observations by light microscopy of treated, unhatched embryos on day 3 postfertilization looked much the same with regard to development as those noted for the previous day treatments. These later-stage embryos, exposed to low (0.1 *μ*M) and medium (1 *μ*M) concentrations of prostratin, were not observably different from those treated with DMSO. As well, exposure to 10 *μ*M prostratin elicited no observable degree of developmental abnormality. This was very much in contrast to specimens treated with 10 *μ*M PMA treatment. As shown in the bottom right panel of Figure 2, this representative PMA-treated embryo was deformed and failed to survive.

**Figure 3:**
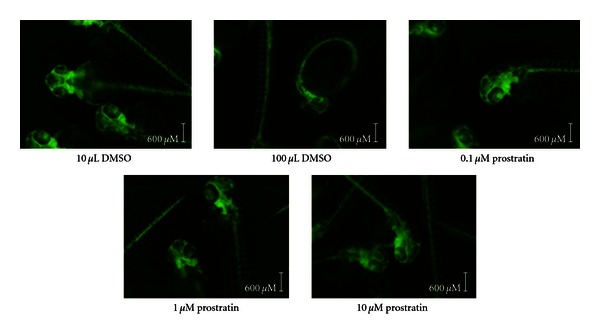
Prostratin treated larvae develop from embryos without obvious gross defects. Embryos, 1-day after fertilization, were treated 3 days consecutively with prostratin at the concentrations as indicated and fluorescent images of specimens captured 24 hours after treatment.

 As shown in Figure 3, overall development of the 4-day postfertilization embryos and newly hatched larvae continued to proceed without observable gross defects with increasing concentrations of prostratin. Note the integrity of the vasculature systems highlighted by eGFP fluorescence. Differences began to emerge shortly after day 3 after fertilization and generally into day 4 when hatching is generally initiated. The most common effects observed were death and/or trapping in the egg sac with exposure to 10 *μ*M PMA.

**Figure 4:**
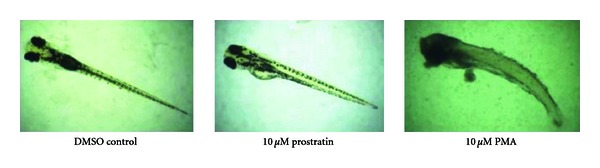
Differential lethal effects of prostratin versus PMA on zebrafish larvae. Zebrafish larvae were treated once with the phorbol esters at the concentrations as indicated. Images were captured 24 hours after treatment.

The concentration-dependent effects of phorbol treatment were next compared on hatched, nontransgenic seven-day-old AB larvae as shown in Figure 4. PMA treatment at 10 *μ*M resulted in rapid and complete lethality as is clearly apparent for the decaying embryo specimen shown to the right side of Figure 4 which was photographed 24 hours after treatment. However, very limited gross morphological deformities were apparent in larvae treated with 10 *μ*M prostratin at least from the time of treatment to visual inspection 24 hours later. Indeed, the 10 *μ*M prostratin-treated larvae appeared morphologically similar to those treated with the DMSO vehicle control (middle and left panels of Figure 4) with high survival rates 24 hours after treatment. These photographed specimens were representative of those in each individually treated population.

### 3.2. Total Protein Expression Is Not Markedly Affected at Sublethal Doses of PKC Modulators

**Figure 5:**
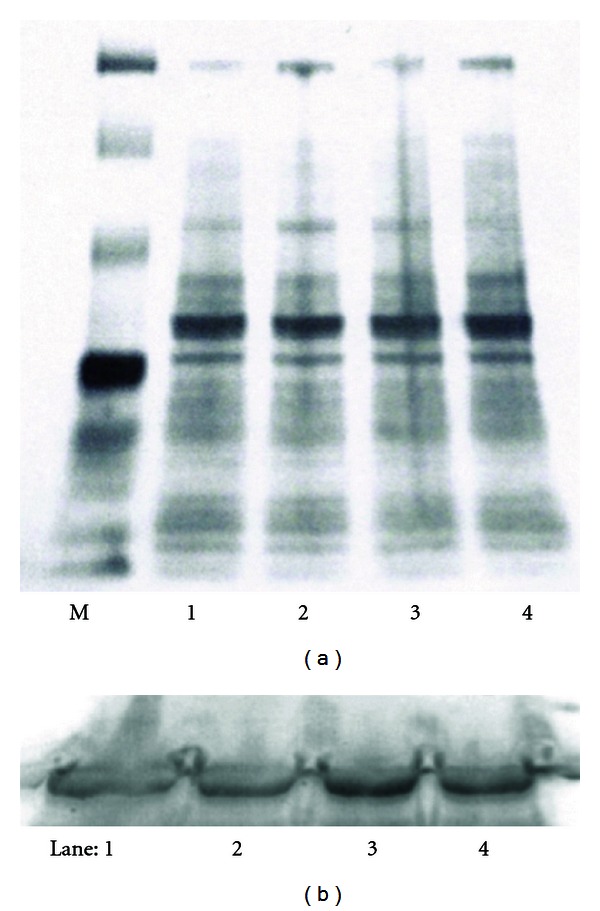
(a) Phorbol ester treatment does not alter global protein expression. Zebrafish larvae were treated with (1) DMSO, (2) 1 *μ*M prostratin, (3) 10 *μ*M prostratin, and (4) 10 *μ*M PMA. Equivalent concentrations of protein samples were subjected to PAGE (b) Phorbol esters upregulate MAPK-8 in treated larvae. Zebrafish larvae were treated with (1) DMSO, (2) 1 *μ*M prostratin, (3) 10 *μ*M prostratin, and (4) 10 *μ*M PMA. Equivalent concentrations of larval lysates were subjected to PAGE/Western analysis using zebrafish specific MAPK-8 antisera.

Protein gel electrophoresis (PAGE) of total proteins from treated larvae was employed to assess any major differences in gene expression as a consequence of phorbol ester exposure. PAGE comparing treated and vehicle control showed no remarkable difference in overall expression among treated or control embryos (data not shown) or larvae as shown in Figure 5(a). Abundant structural proteins in larvae were similar in presence and abundance among all the phorbol-treated larvae samples versus the DMSO control.

### 3.3. Phorbol Treatment Specifically Modulates Intracellular Signaling

PMA, known to be a potent apoptotic mitogen for most cells and tissue systems [15, 16], exhibited the highest degree of inducing developmental defects and lethality relative to prostratin-treated specimens at the same concentration. This suggested agent specificity of action.

 To further demonstrate specificity of action, Western analysis was performed on hatched AB larvae treated with DMSO, prostratin and PMA using MAPK-8 antisera in the expectation that phorbol exposure may alter the expression level of this specific signaling protein. As shown in Figure 5(b), MAPK-8 exhibited modest levels of upregulation following treatment with prostratin or PMA relative to DMSO-control-treated larvae in this representative blot. Western analysis was also employed to assess any changes in the expression of the eGFP protein which remained at similar levels among Fli-1 larvae treated with prostratin or PMA versus DMSO control (data not shown).

### 3.4. Threshold-Specific Dosages of PKC Modulators Correlate with Zebrafish Survival

**Figure 6:**
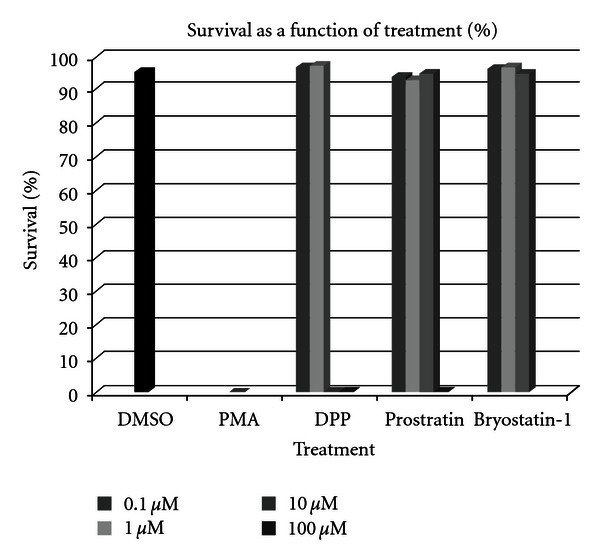
Specific concentrations of PKC modulators induce acute lethality. Zebrafish larvae were treated with the agents at the concentrations as indicated and percent survivors determined 75 minutes after treatment. Each bar represents the mean of four determinations. Standard deviations for each bar are less than 2% of the mean.

The lethal effects of an expanded panel of PKC modulators were next compared in a concentration-dependent fashion using larvae eight days after fertilization. As shown in Figure 6, treatment with different PKC modulators exhibited defined threshold doses for lethality. The phorbol ester, 12-deoxyphorbol-13-phenylacetate (DPP), had a concentration-dependent lethal effect at 10 *μ*M, similar to PMA. Prostratin only elicited rapid death at 100 *μ*M which occurred in the same time frame as dosage with 10 *μ*M PMA and 10 *μ*M DPP, usually within 60–75 minutes after treatment. Of interest is the ability of larvae to tolerate concentrations of bryostatin-1 up to 10 *μ*M. Although 10 *μ*M bryostatin-1 treatment did not induce lethality in the short term, the fish appeared very lethargic 1 hour after treatment and no survivors were observed 24 hours after treatment. The treated fish were unaffected by concentrations of bryostatin-1 at or below 1 *μ*M.

 The survival profiles for each individual compound shown in Figure 6 represent an average of four data sets for treated larvae eight days after fertilization with less than 2% standard deviation from the mean for each set. The lethal effects at the concentrations noted for this larval set also occurred reproducibly for either AB or Fli-1 larvae treated even at earlier postfertilization times. In addition, these survival frequencies were similar for unhatched 4-day postfertilization AB or Fli-1 embryos. (data not shown). Survival frequency for embryos and larvae, regardless of strain was always reproducibly ~94%–99% at nonlethal agent doses, and lethality was consistently very near or at 100% with higher doses of the compounds at all times of treatment beyond three days after fertilization.

## 4. Discussion

The nontumor-promoting phorbol ester prostratin and the lactone bryostatin-1 modulate PKC cascade activity. These agents have been forwarded as potential candidates for the eradication of HAART-persistent cellular reservoirs bearing silent HIV-1 proviral DNA [2–4, 8, 9, 11]. These PKC modulators, including prostratin, upregulate latent viral expression in a number of cell-based systems [2, 8, 9, 11] but have not had widespread clinical use in patients except for bryostatin-1.

 The primary objective of this study was to determine whether treatment with PKC modulators or activators, particularly the phorbol ester prostratin, might elicit a severe cytotoxic profile or induce development effects in tissue systems within a tractable vertebrate model. Each agent elicited a threshold dose for lethality, suggesting specificity of agent action. Specificity was further noted by modest upregulation in the expression of the signaling factor, MAPK-8, within phorbol treated larvae.

 The map kinases (MAPKs) including MAPK-8 (p38) are known to respond to mitogen stimulation, proinflammatory cytokines and environmental stress although this response is primarily mediated through a series of phosphorylation events of preexisting proteins [17, 18]. Our data suggest that some upregulation of MAPK-8 via *de novo* synthesis can occur by exogeneous phorbol treatment of whole zebrafish larvae. This contrasts the expression of the eGFP whose levels remained unchanged in Fli-1 larvae treated similarly with the equivalent concentrations of prostratin and PMA.

 We have some preliminary evidence that PKC modulating compounds induce apoptosis at high doses likely contributing to their obvious lethal effect. This is consistent with the action of phorbol esters and MAPK-8 which can participate in a mitogen-activated cascade to initiate an apoptotic effect [16, 20].

 Assessing the effects of PKC modulators using the zebrafish model are of interest given the ongoing concerns regarding the use of PKC activators or modulators as clinical candidates for administration to humans. This caution may be warranted, since this diverse class of compounds can broadly activate multiple cell-types and can rapidly advance cell-type specific differentiation, maturation, or apoptosis [2, 8, 19]. For instance, prostratin rapidly advances monocyte differentiation [2] and bryostatin-1 induces accelerated maturation of human cord-blood derived dendritic cells [19]. Interestingly, the broad effects of such properties induced by the phorbol ester family are unknown in a whole developing animal model. In contrast, bryostatin-1 has been evaluated clinically at low doses for the treatment of certain human cancers [21–23]. Bryostatin-1 is also regarded as a potential candidate for the treatment of Alzheimer's disease, as it appears that exposure to the compound can extend memory and rescue retrograde or anterograde long-term memory following cerebral ischemia/hypoxia [24, 25].

 The data in this paper might be regarded as encouraging in that low concentrations of PKC modulators, including the phorbol esters, which upregulate latent HIV-1 expression in human cells within a range of 1 to 10 *μ*M [2, 9], showed no obvious effect upon zebrafish embryo or larvae development. In contrast, higher concentrations of PKC activators, elicited almost complete lethality in both zebrafish embryos and early-term larvae.

 Zebrafish embryos and larvae were particularly useful to study the effects of PKC activators or modulators in a whole animal model, since the compounds can be absorbed directly from the media and have clear effects on this organism's development and survival.

 Interestingly, effects on embryos were not observable until after day 3 into day 4 of agent treatment and were most notable with PMA exposure. It is not clear why this very potent tumor-promoting phorbol did not elicit lethal effects on early-stage embryos as quickly as it did for hatched larvae. The chorion may present some barrier toward absorption.

 Zebrafish are recognized as a useful model to rapidly establish paradigms for the investigation and treatment of disabling human diseases. The absorption of exogenous phorbol esters by zebrafish at varying developmental stages appears to be a feasible way to begin to modulate PKC signaling *in vivo* to assess effects on specific tissue systems. Importantly, such processes affected by PKC modulators include, but are not limited to, memory extension and tumorigenesis as noted in other vertebrate systems. [22, 24, 25].

These studies also demonstrate that the nontumor promoting phorbol ester prostratin had no obvious deleterious effects on zebrafish development at concentrations below 10 *μ*M, which is sufficient to upregulate latent HIV-1 expression in human cellular systems [2, 8, 9]. This compound or related agents may deserve further consideration in clinical protocols toward the eradication of HIV-1 latent reservoirs.
